# (2*E*)-1-(4-Chloro­phen­yl)-3-[4-(propan-2-yl)phen­yl]prop-2-en-1-one

**DOI:** 10.1107/S1600536814015281

**Published:** 2014-07-05

**Authors:** Badiadka Narayana, Vinutha V. Salian, Balladka K. Sarojini, Jerry P. Jasinski

**Affiliations:** a Department of Studies in Chemistry, Mangalore University, Mangalagangotri 574 199, India; b Department of Studies in Chemistry, Industrial Chemistry Section, Mangalore University, Mangalagangotri 574 199, India; c Department of Chemistry, Keene State College, 229 Main Street, Keene, NH 03435-2001, USA

**Keywords:** crystal structure

## Abstract

In the title compound, C_18_H_17_ClO, the dihedral angle between the benzene rings is 53.5 (1)°. The mean plane of the prop-2-en-1-one group is twisted by 24.5 (8) and 33.5 (3)° from the chloro- and propanyl-substituted rings, respectively.

## Related literature   

For the non-linear optical properties of the chalcones, see: Sarojini *et al.* (2006 ▶); Poornesh *et al.* (2009 ▶) and for their biological activity, see: Nielsen *et al.* (1998 ▶); Mai *et al.* (2014 ▶); Insuasty *et al.* (2013 ▶). For related structures, see: Jasinski *et al.* (2009 ▶, 2012 ▶); Butcher *et al.* (2007 ▶); Harrison *et al.* (2006 ▶). For standard bond lengths, see: Allen *et al.* (1987 ▶).
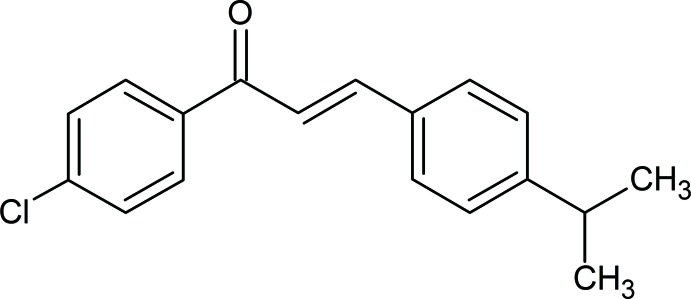


## Experimental   

### 

#### Crystal data  

C_18_H_17_ClO
*M* *_r_* = 284.76
Monoclinic, 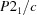

*a* = 8.8547 (5) Å
*b* = 5.8455 (3) Å
*c* = 28.8034 (17) Å
β = 97.396 (6)°
*V* = 1478.46 (14) Å^3^
*Z* = 4
Cu *K*α radiation
μ = 2.21 mm^−1^
*T* = 173 K
0.41 × 0.32 × 0.14 mm

#### Data collection  

Agilent Eos Gemini diffractometer
Absorption correction: multi-scan *CrysAlis PRO* and *CrysAlis RED* (Agilent, 2012 ▶) *T*_min_ = 0.370, *T*_max_ = 1.000
8687 measured reflections
2868 independent reflections
2269 reflections with *I* > 2σ(*I*)
*R*_int_ = 0.023

#### Refinement  

*R*[*F* ^2^ > 2σ(*F* ^2^)] = 0.093
*wR*(*F* ^2^) = 0.286
*S* = 1.04
2868 reflections
183 parameters
H-atom parameters constrained
Δρ_max_ = 0.87 e Å^−3^
Δρ_min_ = −0.44 e Å^−3^

### 

Data collection: *CrysAlis PRO* (Agilent, 2012 ▶); cell refinement: *CrysAlis PRO*; data reduction: *CrysAlis RED* (Agilent, 2012 ▶); program(s) used to solve structure: *SUPERFLIP* (Palatinus *et al.*, 2012 ▶); program(s) used to refine structure: *SHELXL2012* (Sheldrick, 2008) ▶; molecular graphics: *OLEX2* (Dolomanov *et al.*, 2009 ▶); software used to prepare material for publication: *OLEX2*.

## Supplementary Material

Crystal structure: contains datablock(s) I. DOI: 10.1107/S1600536814015281/zs2302sup1.cif

Structure factors: contains datablock(s) I. DOI: 10.1107/S1600536814015281/zs2302Isup2.hkl

Supporting information file. DOI: 10.1107/S1600536814015281/zs2302Isup3.cml

CCDC reference: 1011011

Additional supporting information:  crystallographic information; 3D view; checkCIF report

## Figures and Tables

**Fig. 1.**
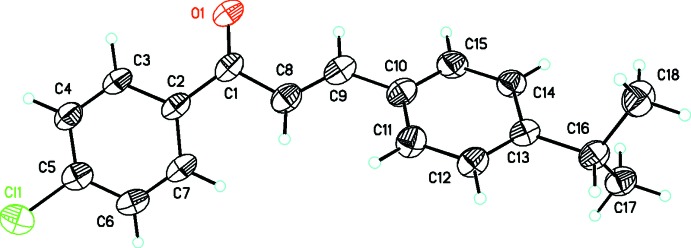
ORTEP drawing of C18H17ClO, showing the atom labeling scheme, with 30% probability displacement ellipsoids.

**Fig. 2.**
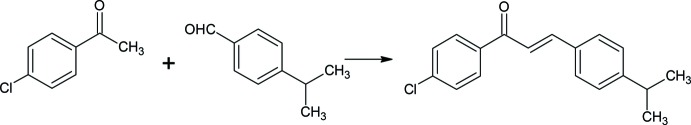
Synthesis of C18H17ClO.

## supplementary crystallographic information

### S1. Comment

Chalcones are an important class of natural compounds and have been widely applied as synthons in synthetic organic chemistry. The nonlinear optical properties of the different chalcone derivatives have been reported (Sarojini *et al.*, 2006; Poornesh *et al.*, 2009). These α,β-unsaturated ketones also possess a wide variety of biological activities, including anti-leishmanial (Nielsen *et al.*, 1998), anticancer (Mai *et al.*, 2014) and antitumor activity (Insuasty *et al.*, 2013). The crystal structures of some chalcone derivatives viz., a second polymorph of (2*E*)-1-(4-fluorophenyl)-3-(3, 4, 5-trimethoxyphenyl)prop-2-en- 1-one, (2*E*)-1-(3,4-dichlorophenyl)-3-(2-hydroxyphenyl)prop-2-en- 1-one (Jasinski *et al.*, 2009, 2012), (2*E*)-1-(2,4-dichlorophenyl)-3-[4-(methylsulfanyl)phenyl] prop-2-en-1-one (Butcher *et al.*, 2007) and 2-bromo-1-chlorophenyl-3-(4-methoxyphenyl) prop-2-en-1-one (Harrison *et al.*, 2006) have been reported. In view of the importance of chalcone derivatives, we report herein the crystal structure of the title compound, C_18_H_17_ClO.

In the title compound, the dihedral angle between the mean planes of the phenyl rings is 53.5 (1)°. The mean plane of the prop-2-en-1-one group (C1/C2/O1/C8) is twisted away from the two phenyl rings by 24.5 (8)° (C2–C7) and 33.5 (3)° (C10–C15) (Fig. 1). Bond lengths are in normal ranges (Allen *et al.*, 1987). No classical hyrogen bonds are observed.

### S2. Experimental

To a mixture of cuminaldehyde (1.5 mL, 0.01 mol) and 4-chloroacetophenone (1.3 mL, 0.01 mol) in ethanol (50 mL), 15 mL of 10 % sodium hydroxide solution was added and stirred at 273–278 K for 3 h (Fig. 2). The precipitate formed was collected by filtration. Single crystals were grown from ethanol by slow the evaporation method (m.p.: 343–345 K).

### S3. Refinement

All of the H atoms were placed in their calculated positions and then refined using the riding model with atom—H bond lengths of 0.95– 1.00 Å or 0.98 Å (CH_3_). Isotropic displacement parameters for these atoms were set to 1.2 (CH) or 1.5 (CH_3_) times *U*_eq_ of the parent atom. The Me group was refined as an ideally rotating group. No twinning has been observed.

### Crystal data

**Table:**

C_18_H_17_ClO	*D*_x_ = 1.279 Mg m^−^^3^
*M**_r_* = 284.76	Melting point = 343–345 K
Monoclinic, *P*2_1_/*c*	Cu *K*α radiation, λ = 1.54184 Å
*a* = 8.8547 (5) Å	Cell parameters from 2529 reflections
*b* = 5.8455 (3) Å	θ = 4.6–72.0°
*c* = 28.8034 (17) Å	µ = 2.21 mm^−^^1^
β = 97.396 (6)°	*T* = 173 K
*V* = 1478.46 (14) Å^3^	Prism, colourless
*Z* = 4	0.41 × 0.32 × 0.14 mm
*F*(000) = 600	

### Data collection

**Table:**

Agilent Eos Gemini diffractometer	2868 independent reflections
Radiation source: Enhance (Cu) X-ray Source	2269 reflections with *I* > 2σ(*I*)
Graphite monochromator	*R*_int_ = 0.023
Detector resolution: 16.0416 pixels mm^-1^	θ_max_ = 72.1°, θ_min_ = 5.0°
ω scans	*h* = −10→10
Absorption correction: multi-scan *CrysAlis PRO* and *CrysAlis RED* (Agilent, 2012)	*k* = −7→6
*T*_min_ = 0.370, *T*_max_ = 1.000	*l* = −35→29
8687 measured reflections	

### Refinement

**Table:**

Refinement on *F*^2^	Primary atom site location: structure-invariant direct methods
Least-squares matrix: full	Hydrogen site location: inferred from neighbouring sites
*R*[*F*^2^ > 2σ(*F*^2^)] = 0.093	H-atom parameters constrained
*wR*(*F*^2^) = 0.286	*w* = 1/[σ^2^(*F*_o_^2^) + (0.1595*P*)^2^ + 1.6733*P*] where *P* = (*F*_o_^2^ + 2*F*_c_^2^)/3
*S* = 1.04	(Δ/σ)_max_ < 0.001
2868 reflections	Δρ_max_ = 0.87 e Å^−^^3^
183 parameters	Δρ_min_ = −0.44 e Å^−^^3^
0 restraints	

### Special details

**Table:**

Geometry. All esds (except the esd in the dihedral angle between two l.s. planes) are estimated using the full covariance matrix. The cell esds are taken into account individually in the estimation of esds in distances, angles and torsion angles; correlations between esds in cell parameters are only used when they are defined by crystal symmetry. An approximate (isotropic) treatment of cell esds is used for estimating esds involving l.s. planes.

### Fractional atomic coordinates and isotropic or equivalent isotropic displacement parameters (Å^2^)

**Table:**

	*x*	*y*	*z*	*U*_iso_*/*U*_eq_	
Cl1	−0.25150 (13)	0.8390 (2)	0.33097 (5)	0.0819 (5)	
O1	0.2335 (4)	0.2755 (5)	0.48768 (11)	0.0695 (8)	
C1	0.2184 (5)	0.4826 (7)	0.48178 (13)	0.0576 (10)	
C2	0.1070 (4)	0.5752 (6)	0.44409 (14)	0.0550 (9)	
C3	0.0602 (5)	0.4451 (7)	0.40431 (15)	0.0625 (10)	
H3	0.1034	0.2981	0.4012	0.075*	
C4	−0.0468 (5)	0.5249 (8)	0.36970 (16)	0.0675 (11)	
H4	−0.0755	0.4358	0.3424	0.081*	
C5	−0.1133 (5)	0.7368 (8)	0.37462 (16)	0.0638 (11)	
C6	−0.0724 (5)	0.8693 (8)	0.41294 (17)	0.0691 (12)	
H6	−0.1192	1.0137	0.4161	0.083*	
C7	0.0386 (5)	0.7896 (7)	0.44713 (16)	0.0677 (12)	
H7	0.0696	0.8833	0.4736	0.081*	
C8	0.3018 (5)	0.6508 (8)	0.51427 (16)	0.0671 (11)	
H8	0.3047	0.8073	0.5055	0.080*	
C9	0.3719 (5)	0.5833 (8)	0.55524 (17)	0.0676 (11)	
H9	0.3598	0.4270	0.5630	0.081*	
C10	0.4664 (5)	0.7227 (8)	0.59013 (16)	0.0652 (11)	
C11	0.5252 (5)	0.9341 (8)	0.57874 (15)	0.0676 (11)	
H11	0.5026	0.9925	0.5478	0.081*	
C12	0.6155 (5)	1.0579 (7)	0.61201 (14)	0.0612 (10)	
H12	0.6546	1.2016	0.6038	0.073*	
C13	0.6505 (4)	0.9774 (6)	0.65722 (13)	0.0529 (9)	
C14	0.5907 (5)	0.7645 (7)	0.66783 (15)	0.0613 (10)	
H14	0.6116	0.7055	0.6987	0.074*	
C15	0.5034 (5)	0.6414 (7)	0.63453 (17)	0.0694 (12)	
H15	0.4671	0.4953	0.6424	0.083*	
C16	0.7492 (5)	1.1144 (7)	0.69393 (15)	0.0614 (10)	
H16	0.7841	1.2548	0.6786	0.074*	
C17	0.6624 (6)	1.1896 (8)	0.73356 (16)	0.0706 (12)	
H17A	0.5750	1.2834	0.7209	0.106*	
H17B	0.7299	1.2794	0.7563	0.106*	
H17C	0.6265	1.0545	0.7490	0.106*	
C18	0.8911 (5)	0.9757 (10)	0.7134 (2)	0.0834 (14)	
H18A	0.8599	0.8406	0.7299	0.125*	
H18B	0.9583	1.0709	0.7351	0.125*	
H18C	0.9454	0.9273	0.6875	0.125*	

### Atomic displacement parameters (Å^2^)

**Table:**

	*U*^11^	*U*^22^	*U*^33^	*U*^12^	*U*^13^	*U*^23^
Cl1	0.0684 (8)	0.0788 (8)	0.1002 (10)	−0.0040 (5)	0.0173 (6)	0.0148 (6)
O1	0.0799 (19)	0.0492 (16)	0.0811 (19)	−0.0082 (14)	0.0161 (15)	−0.0158 (14)
C1	0.064 (2)	0.053 (2)	0.061 (2)	−0.0177 (17)	0.0283 (18)	−0.0123 (17)
C2	0.059 (2)	0.0465 (19)	0.065 (2)	−0.0154 (16)	0.0298 (18)	−0.0121 (16)
C3	0.073 (3)	0.047 (2)	0.071 (2)	−0.0081 (18)	0.022 (2)	−0.0150 (18)
C4	0.081 (3)	0.055 (2)	0.068 (2)	−0.010 (2)	0.016 (2)	−0.0130 (19)
C5	0.059 (2)	0.058 (2)	0.079 (3)	−0.0136 (18)	0.029 (2)	−0.001 (2)
C6	0.066 (3)	0.053 (2)	0.092 (3)	−0.0027 (19)	0.027 (2)	−0.011 (2)
C7	0.077 (3)	0.053 (2)	0.078 (3)	−0.013 (2)	0.028 (2)	−0.022 (2)
C8	0.071 (3)	0.061 (2)	0.073 (3)	−0.011 (2)	0.022 (2)	−0.010 (2)
C9	0.067 (2)	0.059 (2)	0.081 (3)	−0.009 (2)	0.027 (2)	−0.013 (2)
C10	0.065 (2)	0.057 (2)	0.076 (3)	−0.0096 (19)	0.016 (2)	−0.0076 (19)
C11	0.076 (3)	0.064 (3)	0.065 (2)	−0.021 (2)	0.015 (2)	0.0024 (19)
C12	0.066 (2)	0.055 (2)	0.065 (2)	−0.0173 (18)	0.0215 (19)	0.0001 (18)
C13	0.0468 (18)	0.0501 (19)	0.066 (2)	−0.0054 (15)	0.0211 (16)	−0.0031 (16)
C14	0.060 (2)	0.053 (2)	0.071 (2)	−0.0070 (18)	0.0101 (18)	0.0066 (18)
C15	0.072 (3)	0.050 (2)	0.086 (3)	−0.0152 (19)	0.006 (2)	0.013 (2)
C16	0.060 (2)	0.057 (2)	0.069 (2)	−0.0092 (18)	0.0171 (19)	−0.0049 (18)
C17	0.079 (3)	0.066 (3)	0.069 (3)	0.002 (2)	0.020 (2)	−0.008 (2)
C18	0.053 (2)	0.092 (4)	0.105 (4)	−0.004 (2)	0.010 (2)	−0.021 (3)

### Geometric parameters (Å, º)

**Table:**

Cl1—C5	1.743 (5)	C10—C15	1.364 (6)
O1—C1	1.228 (5)	C11—H11	0.9500
C1—C2	1.472 (6)	C11—C12	1.372 (6)
C1—C8	1.486 (6)	C12—H12	0.9500
C2—C3	1.393 (5)	C12—C13	1.382 (6)
C2—C7	1.400 (6)	C13—C14	1.401 (5)
C3—H3	0.9500	C13—C16	1.512 (5)
C3—C4	1.366 (6)	C14—H14	0.9500
C4—H4	0.9500	C14—C15	1.358 (6)
C4—C5	1.386 (6)	C15—H15	0.9500
C5—C6	1.359 (6)	C16—H16	1.0000
C6—H6	0.9500	C16—C17	1.521 (6)
C6—C7	1.379 (7)	C16—C18	1.538 (7)
C7—H7	0.9500	C17—H17A	0.9800
C8—H8	0.9500	C17—H17B	0.9800
C8—C9	1.321 (7)	C17—H17C	0.9800
C9—H9	0.9500	C18—H18A	0.9800
C9—C10	1.469 (6)	C18—H18B	0.9800
C10—C11	1.396 (6)	C18—H18C	0.9800
			
O1—C1—C2	121.0 (3)	C12—C11—H11	119.9
O1—C1—C8	121.9 (4)	C11—C12—H12	119.4
C2—C1—C8	116.9 (4)	C11—C12—C13	121.2 (4)
C3—C2—C1	120.4 (4)	C13—C12—H12	119.4
C3—C2—C7	117.0 (4)	C12—C13—C14	117.6 (4)
C7—C2—C1	122.5 (4)	C12—C13—C16	121.2 (3)
C2—C3—H3	119.4	C14—C13—C16	121.2 (4)
C4—C3—C2	121.3 (4)	C13—C14—H14	119.6
C4—C3—H3	119.4	C15—C14—C13	120.9 (4)
C3—C4—H4	120.2	C15—C14—H14	119.6
C3—C4—C5	119.6 (4)	C10—C15—H15	119.2
C5—C4—H4	120.2	C14—C15—C10	121.6 (4)
C4—C5—Cl1	120.0 (4)	C14—C15—H15	119.2
C6—C5—Cl1	118.7 (4)	C13—C16—H16	108.0
C6—C5—C4	121.3 (4)	C13—C16—C17	112.1 (3)
C5—C6—H6	120.7	C13—C16—C18	110.3 (4)
C5—C6—C7	118.6 (4)	C17—C16—H16	108.0
C7—C6—H6	120.7	C17—C16—C18	110.3 (4)
C2—C7—H7	118.9	C18—C16—H16	108.0
C6—C7—C2	122.1 (4)	C16—C17—H17A	109.5
C6—C7—H7	118.9	C16—C17—H17B	109.5
C1—C8—H8	119.9	C16—C17—H17C	109.5
C9—C8—C1	120.2 (4)	H17A—C17—H17B	109.5
C9—C8—H8	119.9	H17A—C17—H17C	109.5
C8—C9—H9	116.3	H17B—C17—H17C	109.5
C8—C9—C10	127.3 (4)	C16—C18—H18A	109.5
C10—C9—H9	116.3	C16—C18—H18B	109.5
C11—C10—C9	121.8 (4)	C16—C18—H18C	109.5
C15—C10—C9	119.7 (4)	H18A—C18—H18B	109.5
C15—C10—C11	118.5 (4)	H18A—C18—H18C	109.5
C10—C11—H11	119.9	H18B—C18—H18C	109.5
C12—C11—C10	120.3 (4)		
			
Cl1—C5—C6—C7	−179.1 (3)	C8—C9—C10—C11	−16.7 (7)
O1—C1—C2—C3	−25.1 (5)	C8—C9—C10—C15	165.8 (5)
O1—C1—C2—C7	152.0 (4)	C9—C10—C11—C12	−178.8 (4)
O1—C1—C8—C9	−13.1 (6)	C9—C10—C15—C14	179.8 (4)
C1—C2—C3—C4	177.8 (4)	C10—C11—C12—C13	0.1 (7)
C1—C2—C7—C6	−176.0 (4)	C11—C10—C15—C14	2.3 (7)
C1—C8—C9—C10	176.1 (4)	C11—C12—C13—C14	0.1 (6)
C2—C1—C8—C9	162.2 (4)	C11—C12—C13—C16	−179.6 (4)
C2—C3—C4—C5	−1.7 (6)	C12—C13—C14—C15	0.9 (6)
C3—C2—C7—C6	1.1 (6)	C12—C13—C16—C17	115.4 (4)
C3—C4—C5—Cl1	−179.3 (3)	C12—C13—C16—C18	−121.2 (4)
C3—C4—C5—C6	1.1 (6)	C13—C14—C15—C10	−2.1 (7)
C4—C5—C6—C7	0.5 (6)	C14—C13—C16—C17	−64.3 (5)
C5—C6—C7—C2	−1.7 (6)	C14—C13—C16—C18	59.0 (5)
C7—C2—C3—C4	0.6 (6)	C15—C10—C11—C12	−1.3 (7)
C8—C1—C2—C3	159.6 (4)	C16—C13—C14—C15	−179.4 (4)
C8—C1—C2—C7	−23.4 (5)		
